# DDAH1 Promotes Lung Endothelial Barrier Repair by Decreasing Leukocyte Transendothelial Migration and Oxidative Stress in Explosion-Induced Lung Injury

**DOI:** 10.1155/2022/8407635

**Published:** 2022-05-17

**Authors:** Peifang Cong, Changci Tong, Shun Mao, Lin Shi, Xiuyun Shi, Ying Liu, Hongxu Jin, Yunen Liu, Mingxiao Hou

**Affiliations:** ^1^College of Medicine and Biological Information Engineering, Northeastern University, No. 195, Chuangxin Road, Hunnan District, Shenyang, Liaoning Province 110016, China; ^2^Shenyang Medical College, No. 146, Huanghe North Street, Shenyang, Liaoning Province 110034, China; ^3^Emergency Medicine Department of General Hospital of Northern Theatre Command, Laboratory of Rescue Center of Severe Wound and Trauma PLA, No. 83, Wenhua Road, Shenhe District, Shenyang, Liaoning Province 110016, China; ^4^The Second Affiliated Hospital of Shenyang Medical College, The Veterans General Hospital of Liaoning Province, No. 20 Beijiu Road, Heping District, Shenyang, Liaoning Province 110001, China

## Abstract

Explosion-induced injury is the most commonly encountered wound in modern warfare and incidents. The vascular inflammatory response and subsequent oxidative stress are considered the key causes of morbidity and mortality among those in blast lung injury. It has been reported dimethylarginine dimethylaminohydrolase 1 (DDAH1) plays important roles in regulating vascular endothelial injury repair and angiogenesis, but its role in explosion-induced injury remains to be explained. To explore the mechanism of vascular injury in blast lung, 40 C57BL/6 wild type mice and 40 DDAH1 knockout mice were randomly equally divided into control group and blast group, respectively. Body weight, lung weight, and dry weight of the lungs were recorded. Diffuse vascular leakage was detected by Evans blue test. The serum inflammatory factors, nitric oxide (NO) contents, and ADMA level were determined through ELISA. Hematoxylin-eosin staining and ROS detection were performed for histopathological changes. Western blot was used to detect the proteins related to oxidative stress, cell adhesion molecules and leukocyte transendothelial migration, vascular injury, endothelial barrier dysfunction, and the DDAH1/ADMA/eNOS signaling pathway. We found that DDAH1 deficiency aggravated explosion-induced body weight reduction, lung weight promotion, diffuse vascular leakage histopathological changes, and the increased levels of inflammatory-related factors. Additionally, DDAH1 deficiency also increased ROS generation, MDA, and IRE-1*α* expression. Regarding vascular endothelial barrier dysfunction, DDAH1 deficiency increased the expression of ICAM-1, Itgal, Rac2, VEGF, MMP9, vimentin, and N-cadherin, while lowering the expression of occludin, CD31, and dystrophin. DDAH1 deficiency also exacerbated explosion-induced increase of ADMA and decrease of eNOS activity and NO contents. Our results indicated that explosion could induce severe lung injury and pulmonary vascular insufficiency, whereas DDAH1 could promote lung endothelial barrier repair and reduce inflammation and oxidative stress by inhibiting ADMA signaling which in turn increased eNOS activity.

## 1. Introduction

Although explosion-induced injury has long been the most commonly encountered wound in modern warfare, with increasing terrorist incidents, gas explosions and underground explosion events have become more frequent among noncombatants [1]. Explosion can damage tissues, and propelled shell fragments can cause penetrating trauma that is similar to injuries caused by gunshots, fallings, and motor vehicle crashes. Nevertheless, the impacts of overpressure wave that leads to the major blast on the body remain poorly understood [2]. Blast injury is widely known as a high shock rate and high mortality complex injury. It accounts for over 75% of war casualties in the United States military forces [3]. It was also reported that 82% of all injuries caused by terrorists were from bomb blasts, and this number continues to rise [4]. In blast overpressure induced-organ injuries, the lung has been viewed as the primary site that causes morbidity and mortality [5]. Blast overpressure wave disrupts the capillary-alveolar interface, which further induces hemorrhage in the lung and destruction of the alveolar walls. Other pathological findings include hemorrhagic infarcts in the solid abdominal and retroperitoneal organs [6]. Moreover, the severe blast lung will evolve into acute respiratory distress syndrome and impact on the quality of life or prognosis. Accordingly, to investigate, the mechanism of explosion-induced lung injury will be of great significance for the treatment of wounded individuals.

Inflammatory responses have been shown to be activated in explosion-induced injury [7]. In our previous study, proteomic analysis helped confirm that blast lung injury induced early inflammatory response through regulating the expression of key proteins involved in the inflammatory process [8]. Additionally, expression levels of leukocyte adhesion- (LA-) related proteins were increased by blast exposure. This is a characteristic feature of vascular inflammation, which is strongly associated with acute lung injury [9]. LA in the vascular endothelium is reportedly regulated via cell adhesion molecules (CAMs), whereas increased plasma levels of CAMs have been documented in ischemia-reperfusion injury [10]. In addition, endothelial integrins such as intercellular adhesion molecule (ICAM), soluble vascular cell adhesion molecule (sVCAM), proinflammatory cytokine, and monocyte chemoattractant protein-1 (MCP-1) were significantly increased in brain blast injury tissues compared with the control group [11]. The vascular inflammatory response and subsequent oxidative stress are considered to be the key causes of morbidity and mortality in acute lung injury. Several studies elucidated that explosion-induced vascular inflammation caused increased inflammatory cytokine production and leukocyte infiltration and injury, whereas CAM upregulation was reported to be controlled through inflammatory molecules, including IL-1*β*, TNF-*α*, and IFN-*γ* [12, 13]. However, the specific mechanism of vascular dysfunction in blast lung injury has not been clarified.

Dimethylarginine dimethylaminohydrolase 1 (DDAH1) is essential in regulating vascular endothelial injury repair and angiogenesis, and it is also important in degrading asymmetric dimethylarginine (ADMA) to maintain nitric oxide (NO) signaling. DDAH1 deficiency results in significantly increased ADMA levels, accelerated cell oxidation, and apoptosis [14]. ADMA, an endogenous nitric oxide synthase (NOS) inhibitor, is related to hypertension, diabetes, cardiac dysfunction, vessel injury, and other cardiovascular diseases. It is reported that ADMA attenuates endothelial nitric oxide synthase (eNOS) activity to reduce NO production and also leads to NOS uncoupling to generate free radicals [15]. eNOS, a homodimer, is predominantly located within the vascular endothelium and cardiac myocytes, as well as the renal epithelium. NO is a highly reactive signaling molecule, with strong vasodilatory, anti-inflammatory, and antioxidant properties. Reddy et al. found that overexpression of DDAH1 could significantly promote the proliferation of endothelial cells by reducing ADMA levels, increasing NO production, and inducing expression of CD31, VEGF, and HIF-1*α* [16]. Additionally, another study showed that irbesartan attenuates indomethacin-induced mucosal injury by increasing the expression of DDAH1 while decreasing matrix metalloproteinase-9 (MMP9), TNF-*α*, COX-2, caspase-3, and ADMA in gastric mucosa cells [17]. However, the effects of DDAH1/ADMA/eNOS signaling on explosion-induced lung injury have not been studied. Therefore, we hypothesize that explosion-induced lung injury will result in decreased DDAH1 levels and increased ADMA levels to restrict vascular injury repair and aggravate inflammation and oxidative stress.

## 2. Materials and Methods

### 2.1. Animal and Experimental Protocols

Forty DDAH1 knockout (DDAH1 KO) mice were provided by Jackson Laboratory (Sacramento, CA), and the same amount of C57BL/6 wild-type (WT) mice was purchased from the Beijing Vital River Laboratory Animal Technology Limited Company, P.R. China. All mice were maintained in a room with a temperature of 20 ± 2°C, humidity of 55–65%, and free access to food and water. After acclimation, wild type and DDAH1 KO were exposed to explosion-induced injury, and samples were collected at 24 hours. Animal welfare and experimental design were approved by the Ethics Committee of the General Hospital of Northern Theater Command.

### 2.2. Lung Blast Injury

Explosion-induced lung injury was established as previously described [18]. Briefly, aluminum foils were fixed in the middle layer. After anesthetized by 2% pentobarbital sodium (intraperitoneal injection, 1.5 ml/kg), mice were put on the protective device with their chest exposed. The air pressure in the lower part of the device was increased through a pressure pump until the burst of aluminum foil. Shock waves, generated when compressed air was rapidly diffused, were directed at the chest of the mice and recorded by a computer through a pressure sensor and data cable. The formula of pressure waveform was as follows: pressure (PSI) = voltage value∗1000/50.08. In this experiment, the instantaneous overpressure was 321 ± 24 PSI.

### 2.3. Sample Collection and Processing

After 12 h of fasting and 4 h of water deprivation preoperatively, mice were intraperitoneally anesthetized as described above. Serum was collected, and the lung tissue was weighed. The left lobe was immersed in 10% formalin buffer to detect histological changes. The upper lobe of the right lung was weighed to get the wet weight, and the dry weight was obtained through drying the tissue in an oven at 58°C for 8 h. Calculate the ratio of wet weight to dry weight to evaluate edema formation. The rest of the fresh lung tissue of each mouse was refrigerated at -80°C for protein determination.

### 2.4. Enzyme-Linked Immunosorbent Assay (ELISA)

According to the manufacturers' instruction, levels of ADMA, NO, and inflammatory factors, such as IL-1*β*, IL-6, and TNF-*α* in the plasma of mice were detected by ELISA kits (Nanjing Jiancheng Bioengineering Institute, Nanjing, People's Republic of China). Microplate reader was used to read OD at 450 nm. A standard curve was generated, and curve equation was calculated to determine the concentrations of the samples.

### 2.5. Evan's Blue Test

Evan's blue test was done as previously described [19]. Mice were injected with 2% Evans blue (2 mL/kg) through tail vein 30 min prior to sacrifice. After anesthesia, the chest was opened, the right atrium was then cut, and intrahepatic perfusion with 100 mL of heparin saline (0.9% sodium chloride 20 U/mL heparin sodium) was carried out. The lung tissue was weighed and sheared into smaller pieces before being placed in 1 mL N,N-dimethylformamide (DMF) at 60°C for 24 h. The tissue block was homogenized and centrifuged at 12,000 r/min for 20 min. Microplate reader was used to detect the standard curve at 630 nm absorbance, and the calculation was subsequently performed.

### 2.6. Histological Analysis

The left lungs fixed in 10% formaldehyde were embedded in paraffin blocks by Leica Microsystem tissue processor (ASP 300S, Germany). Before hematoxylin and eosin (H&E) staining, paraffin blocks were sliced into sections of 3 *μ*m thickness by a Leica Microsystem microtome (Model RM 2265, Germany).

### 2.7. Reactive Oxygen Species (ROS) Detection

2,3-Dimethoxy-1,4-naphthoquinone (1 : 100; cat. no. D5439; Sigma, USA) was used to stain lung tissue sections. After twenty minutes of staining, sections were observed and photographed under a fluorescence microscope (Olympus, Japan).

### 2.8. Western Blotting

Western blotting was performed as previously described [18]. Briefly, lung tissues were lysed, and the protein concentrations of tissue were measured. Equal amounts of samples were transferred onto a polyvinylidene fluoride membrane after protein separation on polyacrylamide gels was conducted. The membranes were blocked in 5% skim milk PBST buffer and were incubated in the appropriate primary antibody (Supplementary Table 1) overnight at 4°C and then in corresponding horseradish peroxidase-labeled secondary antibody (Supplementary Table 2) for 1.5 h at room temperature. Proteins were visualized with Western enhanced chemiluminescence substrate (Bio-Rad Laboratories, Inc., Hercules, CA, USA) and a Tanon 5200 full automatic chemiluminescence image analysis system (Tanon Science and Technology Co., Ltd., Shanghai, China).

### 2.9. Statistical Analysis

Mean ± standard deviation was used to express the data. Analyses were performed using SPSS 20.0 statistical software. Measurement data were analyzed using the *t*-test and analysis of variance. All statistical tests were two-tailed probability tests. A value of *P* < 0.05 was regarded as significant.

## 3. Results

### 3.1. Explosion-Induced Severe Lung Injury and Leukocyte Infiltration Are Exacerbated in DDAH1 KO Mice

**Figure 1:**
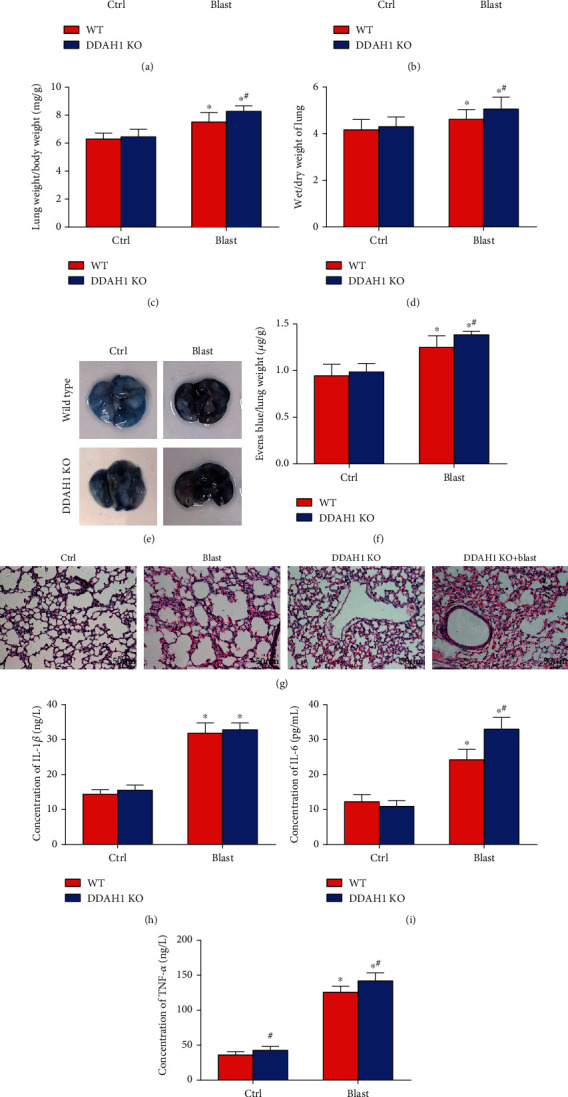
Lung inflammation increased in the lung tissue after explosion. (a) Body weight of each group. (b) Lung weight of each group. (c) Lung weight/body weight of each group. (d) Wet/dry weight of lung in WT mice and DDAH1^−/−^ mice. (e) Evans blue dye of lung in WT mice and DDAH1^−/−^ mice. (f) Evans blue/lung weight of each group. (g) HE staining of the lung in WT mice and DDAH1^−/−^ mice after explosion. (h)–(j) The concentration of inflammatory factors detected in the serum by ELISA. Data are mean ± SD. ∗*P* < 0.05, compared with the same kind of mice in the control group, ^#^*P* < 0.05, compared with C57BL/6 wild-type mice in the same group.

After the blast explosion, the average body weight of WT mice decreased significantly, whereas the lung weight, ratio of lung weight to body weight, and ratio of wet weight to dry weight significantly increased, compared with the control group. In DDAH1 KO mice, the degree of change in these indexes was exacerbated after blast explosion (Figures 1(a)–1(d), *P* < 0.05).

The Evan's blue test showed pulmonary vascular breakdown after explosion, and vascular rupture caused the leakage of Evan's blue dye into the lung tissue after explosion. The degree of diffuse vascular leakage worsened in the DDAH1 KO mice (Figures 1(e) and 1(f), *P* < 0.05).

Leukocyte infiltration was observed in the blast group, which was significantly exacerbated in the DDAH1 KO mice. (Figure 1(g), *P* < 0.05). Serum IL-1*β*, TNF-*α*, and IL-6 levels peaked after blast explosion, while they were further increased in DDAH1 KO mice (Figures 1(h)–1(j), *P* < 0.05).

### 3.2. DDAH1 KO Exacerbates Explosion-Induced Lung Oxidative Stress

**Figure 2:**
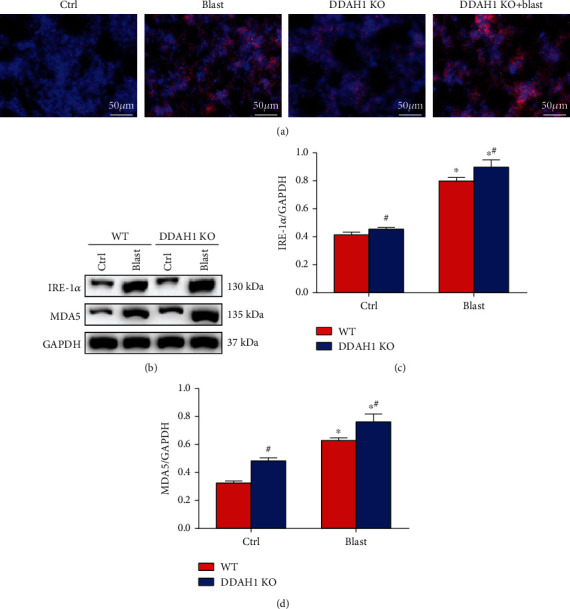
Oxidative stress in the lung tissue after explosion. (a) ROS generation of lung tissue in WT mice and DDAH1^−/−^ mice. (b) Western blot of oxidant enzymes in each group. (c) Relative density of IRE-1*α*. (d) Relative density of MDA. Data are mean ± SD. ∗*P* < 0.05, compared with the same kind of mice in the control group, ^#^*P* < 0.05, compared with WT mice in the same group.

To determine the level of explosion-induced lung oxidative stress in WT and DDAH1 KO mice, ROS production was detected, and inositol-requiring enzyme-1*α* (IRE-1*α*) and malondialdehyde (MDA5) protein levels were investigated by western blotting. Our data demonstrated that explosion clearly increased the generation of ROS, MDA5, and IRE-1*α* expression, whereas DDAH1 KO significantly increased explosion-induced ROS generation and MDA5 and IRE-1*α* protein levels (Figure 2, *P* < 0.05).

### 3.3. DDAH1 KO Enhances Explosion-Induced Changing of Cell Adhesion Molecules (CAMs) and Leukocyte Transendothelial Migration

**Figure 3:**
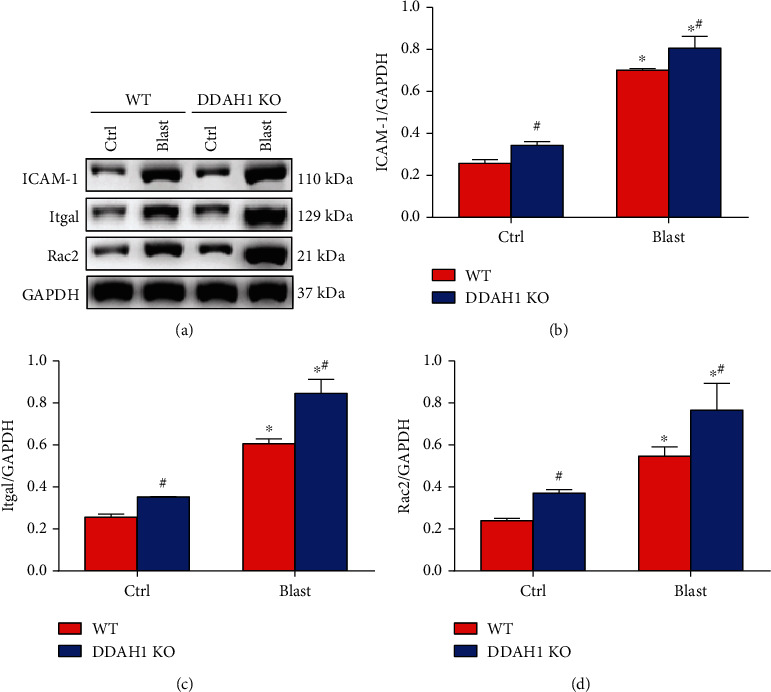
Expression of CAMs and leukocyte transendothelial migration-related proteins in the lung tissue after explosion. (a) Western blot of CAMs and leukocyte transendothelial migration-related proteins in each group. (b) Relative density of ICAM-1. (c) Relative density of Itgal. (d) Relative density of Rac2. Data are mean ± SD. ∗*P* < 0.05, compared with the same kind of mice in the control group, ^#^*P* < 0.05, compared with WT mice in the same group.

Because of the critical effects of CAMs in leukocyte transendothelial migration, we explored how explosion affected the expression of inflammatory molecules and CAMs in both WT and DDAH1 KO mice. CAM expression levels, including intercellular adhesion molecule-1 (ICAM-1), were markedly increased in the lungs of the WT mice after explosion, and this upregulation was aggravated in explosion-induced DDAH1 KO mice (*P* < 0.05). Our results also demonstrated that explosion could clearly promote the expression of leukocyte transendothelial migration-related proteins integrin subunit alpha L (Itgal) and Ras-related C3 botulinum toxin substrate 2 (Rac2) in lung tissue, while DDAH1 KO significantly enhanced explosion-induced increased levels of Itgal and Rac2 (Figure 3, *P* < 0.05). These results suggest that DDAH1 KO upregulates explosion-induced myeloid cell infiltration and CAM expression.

### 3.4. DDAH1 KO Aggravates Blast Explosion-Induced Lung Vascular Injury

**Figure 4:**
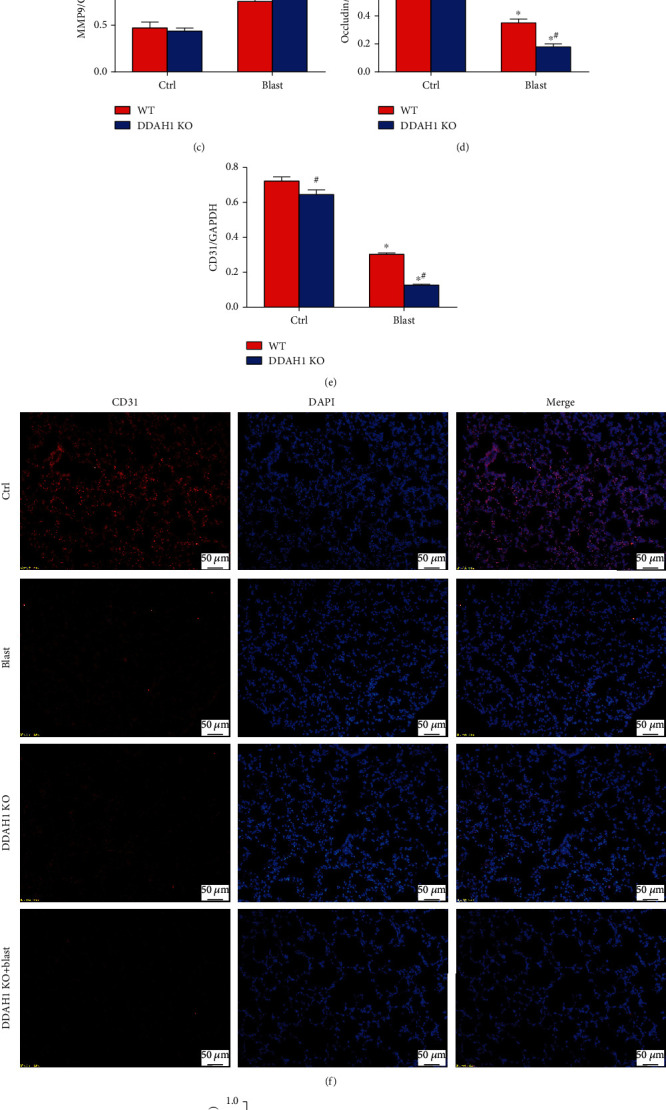
Expression of vascular injury-related proteins in the lung tissue after explosion. (a) Western blot of vascular injury-related proteins in each group. (b) Relative density of VEGF. (c) Relative density of MMP9. (d) Relative density of Occludin. (e) Relative density of CD31. (f) Immunofluorescence of CD31. (g) Positive cell rate of CD31 in immunofluorescence. Data are mean ± SD. ∗*P* < 0.05, compared with the same kind of mice in the control group, ^#^*P* < 0.05, compared with WT mice in the same group.

Our results showed that explosion could induce significant increases in VEGF and MMP9 expression, while occludin and CD31 levels tended to decrease in WT mice. DDAH1 KO significantly aggravated explosion-induced increases in VEGF and MMP9 expression and decreases of occludin and CD31 expression (Figures 4(a)–4(e), *P* < 0.05). Immunofluorescence analysis of CD31 also demonstrated the same trend observed in western blot analysis (Figures 4(f) and 4(g), *P* < 0.05). Our data demonstrated that DDAH1 KO could delay explosion-induced lung vascular injury repair and angiogenesis.

### 3.5. DDAH1 KO Aggravates Blast Explosion-Induced Endothelial Barrier Dysfunction

**Figure 5:**
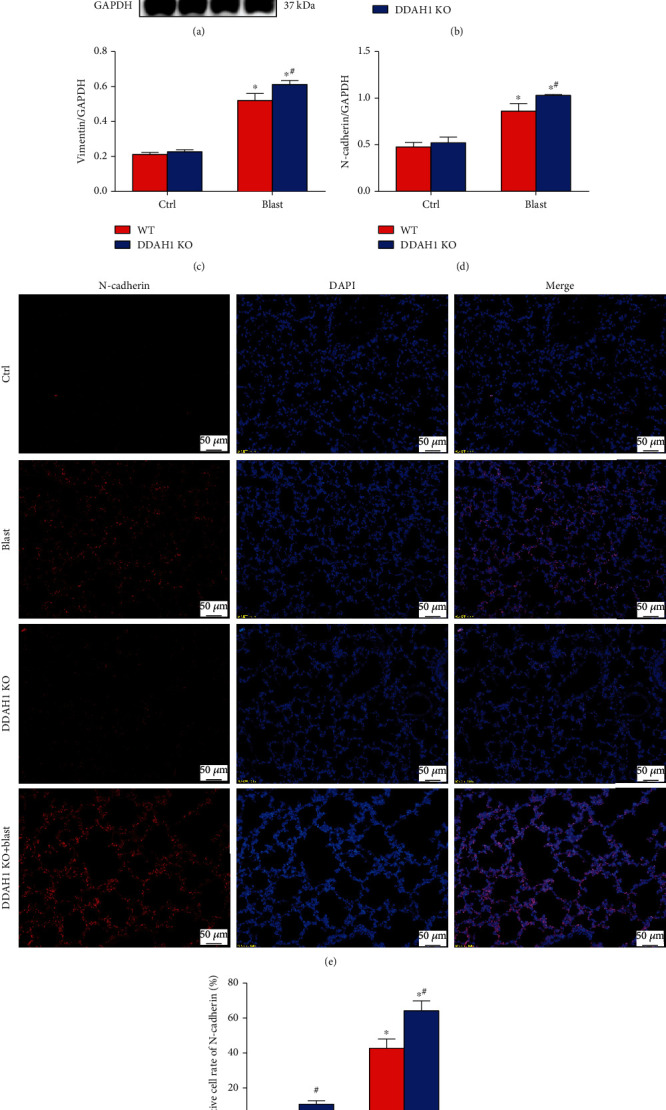
Expression of endothelial barrier dysfunction related proteins in the lung tissue after explosion. (a) Western blot of endothelial barrier dysfunction related proteins in each group. (b) Relative density of dystrophin. (c) Relative density of Vimentin. (d) Relative density of N-cadherin. (e) Immunofluorescence of N-cadherin. (f) Positive cell rate of N-cadherin in immunofluorescence. Data are mean ± SD. ∗*P* < 0.05, compared with the same kind of mice in the control group, ^#^*P* < 0.05, compared with WT mice in the same group.

Our results showed that explosion significantly decreased dystrophin levels, but increased vimentin and N-cadherin levels in WT mice. DDAH1 KO significantly aggravated the explosion-induced decrease of dystrophin expression and increase of vimentin and N-cadherin (Figure 5, *P* < 0.05).

### 3.6. DDAH1 KO Aggravates Explosion-Induced Changes to the DDAH1/ADMA/eNOS Signaling Pathway

**Figure 6:**
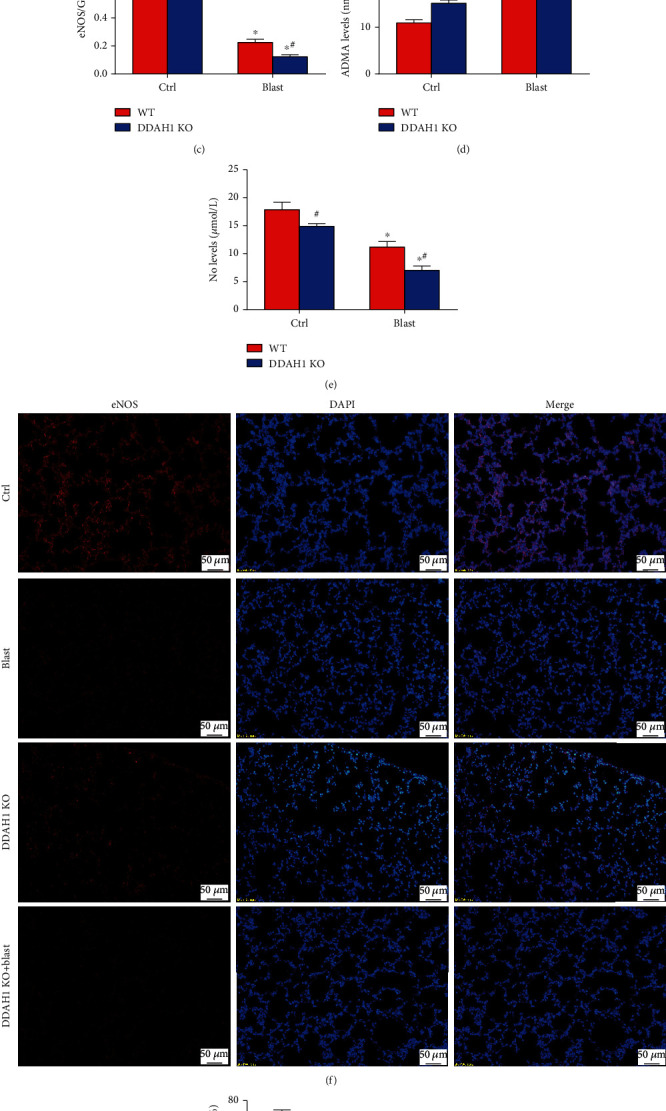
DDAH1 KO aggravated blast explosion-induced changing in DDAH1/ADMA/eNOS signaling pathway. (a) Western blot of DDAH1/ADMA/eNOS signaling pathway in each group. (b) Relative density of DDAH1. (c) Relative density of eNOS. (d) ADMA levels in the lung tissue. (e) NO contents in serum. (f) Immunofluorescence of eNOS. (g) Positive cell rate of N-cadherin in immunofluorescence. Data are mean ± SD. ∗*P* < 0.05, compared with the same kind of mice in the control group, ^#^*P* < 0.05, compared with WT mice in the same group.

Explosion could induce a significant reduction in DDAH1 and eNOS expression and a significant increase of ADMA contents in the lung of WT mice. The ADMA increase was higher in explosion-induced DDAH1 KO mice, which in turn inhibited eNOS expression levels (Figures 6(a)–6(d), *P* < 0.05). NO contents and immunofluorescence of eNOS also showed the same trend seen with western blot analysis of eNOS (Figures 6(e)–6(g), *P* < 0.05). Explosion also caused a slight increase on the expression of iNOS, but not significantly (Supplementary Figure 1, *P* > 0.05).

## 4. Discussion

Blast lung injury is one of numerous injuries resulting from an overpressure shock wave. It has many pathophysiological consequences, including a spectrum of adverse cardiovascular and respiratory effects [20]. Thus, blast overpressure wave can cause a fatal primary blast injury syndrome, where lung is most susceptible to these injuries. In this study, our major findings are that exposure of thorax to blast wave could induce the following: (i) lung injury, presenting as pulmonary edema with increased leukocyte infiltration; (ii) inflammation, as indicated by increased serum levels of inflammatory factors and leukocyte transendothelial migration; (iii) an oxidative stress response, as suggested by ROS production and the expression of oxidant enzymes; (iv) pulmonary vascular injury, as indicated by pulmonary vascular leakage and the expression of endothelial barrier dysfunction related proteins; and (v) inhibition of DDAH1 and activation of ADMA signaling, which lead to reduced eNOS levels. All of these observed effects were aggravated by DDAH1 deficiency. These findings indicated that thoracic explosion could induce inflammation and pulmonary vascular endothelial barrier dysfunction via the DDAH1/ADMA/eNOS signaling pathway.

Explosion is recognized to cause lung injury through promoting the expression level of inflammatory factors and potentially contribute to leukocyte transendothelial migration in the lung. In this study, we found that explosion could lead to severe lung injury caused by inflammation and leukocyte infiltration. Li et al. demonstrated that blast overpressure remarkably promoted the production of systemic inflammatory-related cytokines, and it also triggered obvious pulmonary edema and inflammation in the lungs [21]. In addition, Barnett-Vanes et al. showed that a significant increase of neutrophils and CD43Lo/His48Hi monocytes in the blood stream, along with increased circulating proinflammatory chemo/cytokines, KC, and IL-6, was observed in rats that were placed under blast waves for a longer duration [22]. As CAMs are essential in the regulation of vascular homeostasis and innate and adaptive immune responses, their role in inflammation is significant. According to structural features and functions, CAMs can be divided into five major groups and have multiple functions in diseases related to inflammation, immunity, allergy, and neoplastic conditions [23]. VCAM-1 and ICAM-1 are related to the pathophysiological process of vascular inflammation. VCAM-1, ICAM-1, and E-selectin were reportedly upregulated in an experimental sepsis model of human umbilical vein endothelial cells [24]. ICAM-1 mRNA can also interact with Itgal, which mainly encodes a key T cell integrin, lymphocyte function-associated antigen 1(LAF1), and is considered to be an important factor in regulating T cell activation and migration [8]. Itgal also plays important roles in recruiting cells to sites of inflammation and has downstream effects on cellular signaling [25]. Rac2, a member of the Rho family, is associated with neutrophil chemotaxis, leukocytosis, oxidative stress, and adherent junction [26]. Rac-mediated ROS generation is involved in multiple pathophysiologic processes, including endothelial damage and leukocyte transendothelial migration [27]. During inflammation, Rac2 signaling could progress by clustering ICAM-1. In our study, the expression of ICAM-1, Itgal, and Rac2 was markedly elevated in the lung after explosion, which contributed to leukocyte transendothelial migration and inflammation (Figure 3). Additionally, the above changes also mediated oxidative stress in the lung (Figure 2).

A healthy pulmonary endothelial barrier is indispensable in the regulation of the exosmosis of cells and cytokines from the blood and the sustainment of the stability of the intravascular environment. The destruction of adhesion and connection of vascular endothelial cells are the main determinant of edema formation and inflammatory cell infiltration, and it is also the key factor of acute lung injury and acute respiratory distress syndrome [28]. Endothelial barrier dysfunction in blast explosion-induced lung injury was observed in our previous study, but the specific mechanism has not been explored. Pulmonary vascular endothelial barrier dysfunction could be caused by various factors, such as upstream signaling and inflammation or wound. It can manifest as a variety of changes in endothelial dysfunction-related proteins, such as MMP-9, VEGF, CD31, occludin, ZO-1, and vascular endothelial cadherin [29–31]. VEGF is a highly specific vascular endothelial cell mitogen and survival factor, and its overexpression disrupts the lung architecture during normal lung development. VEGF can activate MMP9 expression, which has been shown to disrupt the tight junction proteins in inflammatory diseases of the central nervous system [32]. In the process of inflammation-induced endothelial barrier dysfunction, the epithelial-mesenchymal transition (EMT) is an important pathophysiological process, the biological process where epithelial cells convert into cells with a mesenchymal phenotype through cell-biological programs [33]. As mesenchymal markers, N-cadherin and vimentin often increase during EMT. This is contributed to many respiratory diseases, including asthma, idiopathic pulmonary fibrosis, and chronic obstructive pulmonary disease [34]. Yang et al. found that the expression levels of vimentin and *α*-smooth muscle actin increased in human lung epithelial cells under mechanical ventilation injury [35]. Dystrophin is a large protein in which mutations can result in progressive muscle weakness, respiratory distress, and cardiovascular disorder [36–38]. It attaches the extracellular matrix to the cytoskeleton through F-actin and participates in events of signaling and synaptic transmission. Morici et al. found that a lack of dystrophin might impair the bronchial epithelium repair [37]. In this study, Evan's blue dye and the observed upregulation of VEGF, MMP9, N-cadherin, and vimentin demonstrated the increase of pulmonary vascular permeability and the occurrence of EMT. Furthermore, downregulation of occludin, CD31, and dystrophin suggested that pulmonary endothelial dysfunction occurred after blast explosion (Figures 1, 4, and 5).

The damage resulting from DDAH activity can lead to an increased ADMA concentration and a series of reactions, such as severe inflammation and oxidative stress, whereas overexpression of DDAH1 could reduce injury. Chandra et al. demonstrated that DDAH1 overexpression could enhance wound repair in agricultural organic dust-treated airway epithelial cells [39]. Similar results were shown by Kinker et al. who found that DDAH1 expression declined in the lung tissue following house dust mite exposure. It was indicated that the decreased DDAH1 expression is associated with elevated ADMA levels in BALF and serum. However, DDAH1 overexpression caused a significant decrease in total cell and eosinophil numbers, suggesting that DDAH1 overexpression can attenuate allergen-induced airway inflammation [40]. Another study previously showed that higher plasma ADMA concentrations were detected in preterm infants. This could induce inhibition of NO synthesis and deteriorated pulmonary function, suggesting that higher ADMA levels are correlated with poor pulmonary functions in preterm infants [41]. With regards to leukocyte transendothelial migration, eNOS could decrease the expression of macrophage chemoattractant protein-1, which limits leukocytes' trafficking to endothelium. Koudelka et al. demonstrated that decreasing ADMA levels and recovering eNOS expression could block the expression of IL-6 and ICAM-1, which improved the observed endothelial dysfunction and inflammatory processes in the lungs [42]. Deficiency of eNOS could also aggravate the infiltration of Itgal-positive white blood cells in lung tissue of ischemia-reperfusion (I/R) injury. Improvement in eNOS activity and higher DDAH1 protein expression levels were believed to have a positive effect on inflammation and oxidative stress in pulmonary hypertension [43]. In this study, our data confirmed that DDAH1 KO could exacerbate explosion-induced lung injury through increasing lung leukocyte infiltration and proinflammatory cytokine expression, as well as elevating the expression of CAMs (Figures 1 and 3). Our findings indicated that explosion-induced severe lung injury and leukocyte infiltration were associated with increased ADMA levels and decreased levels of DDAH1, eNOS activity, and NO contents (Figure 6).

DDAH1 promotes vascular injury repair and angiogenesis, whereas ADMA is strongly associated with endothelial dysfunction, hypertension, and ischemia-reperfusion injury [44, 45]. It is also widely recognized that DDAH1 is the upstream protein that inhibits ADMA and triggers eNOS to produce NO. eNOS is one of three main NOS isoforms that regulate NO levels. It is highly expressed in vascular endothelial cells, and its activity is inhibited by ADMA. NO is a ubiquitous signaling molecule within the cardiovascular system that promotes endothelial cell proliferation and migration. In vascular cells, it is mainly produced by eNOS as a critical factor of vascular homeostasis. NO deficiency is one of the leading factors that cause endothelial dysfunction. Zhao et al. found that DDAH1 had a protective role for the blood-brain barrier by preventing tight junction protein degradation through decreasing ADMA levels and increasing NO levels in ischemic stroke [46]. Several documents revealed that NO production and increased VEGF expression levels may play critical roles in lung vascular injury repair and angiogenesis [47–50]. Recent studies suggested that activating the eNOS-VEGF signaling pathway could promote angiogenesis via enhancing the viability, migration, and tube formation of endothelial cells [51]. Garbincius et al. found that DDAH1 overexpression could increase exercise tolerance via promoting NO signaling and partially restoring dystrophin expression in patients with Duchenne muscular dystrophy [52]. Additionally, a deficiency in the eNOS signaling pathway could exacerbate peritoneal fibrosis in mice by increasing the expression of vimentin. In this study, we observed that pulmonary endothelial dysfunction was aggravated in DDAH1 KO mice, which indicated that DDAH1 was important in lung vascular injury repair (Figures 4 and 5).

**Figure 7:**
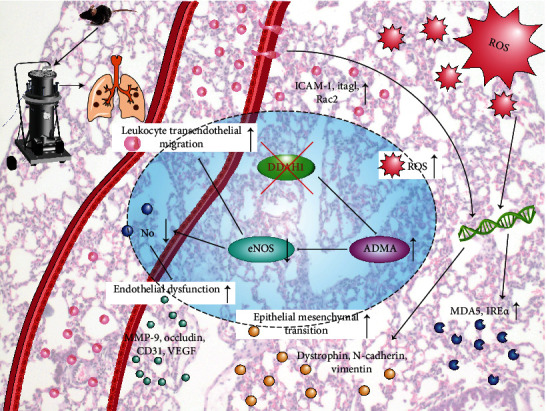
DDAH1 deficiency retards lung endothelial barrier repair by increasing leukocyte transendothelial migration and oxidative stress in explosion-induced lung injury.

From the abovementioned findings, we could infer that explosion can exert its adverse effects on endothelial cell function by downregulating DDAH1 and accumulating ADMA, resulting in lung injury. The absence of DDAH1 caused increased ADMA levels, which led to decreased eNOS and NO contents and increased generation of ROS, thereby leading to leukocyte transendothelial migration, oxidative stress, endothelial dysfunction, and EMT (Figure 7). All these changes demonstrated that DDAH1 might be a major factor in pulmonary endothelial dysfunction injury caused by explosion.

## 5. Conclusions

In summary, our data showed that explosion can induce severe lung injury, leukocyte infiltration, and pulmonary vascular insufficiency, whereas DDAH1 could promote lung endothelial barrier repair and reduce inflammation and oxidative stress by degrading ADMA signaling and increasing eNOS activity. Given the role of DDAH1 in protection against such injuries, our findings suggested that activating DDAH1-related signaling pathways may be able to provide insight for designing new therapies for the management of explosion-associated lung injury complications.

## Data Availability

The data used to support the findings of this study are included within the article.

## Supplementary Materials

Supplementary Materials Supplementary Table 1: primary antibody list. Supplementary Table 2: secondary antibody list. Supplementary Figure 1: expression of iNOS in the lung tissue after blast exposure. (a) Western blot of iNOS in each group. (b) Relative density of iNOS. Data are mean ± SD.
