# Supplementary material for: DDAH1 Promotes Lung Endothelial Barrier Repair by Decreasing Leukocyte Transendothelial Migration and Oxidative Stress in Explosion-Induced Lung Injury
Source: Oxid Med Cell Longev. 2022 May 17;2022:8407635. doi: 10.1155/2022/8407635 (PMC9130000; doi:10.1155/2022/8407635)
Supplement: Supplementary Materials — Supplementary Table 1: primary antibody list. Supplementary Table 2: secondary antibody list. Supplementary Figure 1: expression of iNOS in the lung tissue after blast exposure. (a) Western blot of iNOS in each group. (b) Relative density of iNOS. Data are mean ± SD. [file 8407635.f1.zip › 8407635.f1/supplementary table and figure.docx]

Supplementary Table 1. Primary Antibody List

|  | Dilution ratio | Catalogue number | Company |
| --- | --- | --- | --- |
| IRE-1α | 1:1000 | #3294 | cell signaling technology |
| MDA | 1:1000 | #5321 | cell signaling technology |
| VEGF | 1:1000 | ab214424 | Abcam |
| CD31 | 1:1000 | ab222783 | Abcam |
| MMP9 | 1:1000 | ab38898 | Abcam |
| Occludin | 1:1000 | ab216327 | Abcam |
| Dystrophin | 1:1000 | ab275391 | Abcam |
| Vimentin | 1:1000 | ab92547 | Abcam |
| N-Cadherin | 1:1000 | #14215 | cell signaling technology |
| ICAM1 | 1:1000 | ab222736 | Abcam |
| Itgal | 1:1000 | ab186873 | Abcam |
| Rac2 | 1:1000 | ab2244 | Abcam |
| DDAH1 | 1:1000 | ab180599 | Abcam |
| ADMA | 1:1000 | #13522 | cell signaling technology |
| eNOS | 1:1000 | #32027 | cell signaling technology |
| iNOS | 1:1000 | #13120 | cell signaling technology |
| GAPDH | 1:5000 | #2118 | cell signaling technology |

Supplementary Table 2. Secondary Antibody List

|  | Dilution ratio | Catalogue number | Company |
| --- | --- | --- | --- |
| anti-mouse secondary antibody | 1:4000 | #7076 | cell signaling technology |
| anti-rabbit secondary antibody | 1:4000 | #7074 | cell signaling technology |
| anti-goat secondary antibody | 1:4000 | ab6741 | Abcam |


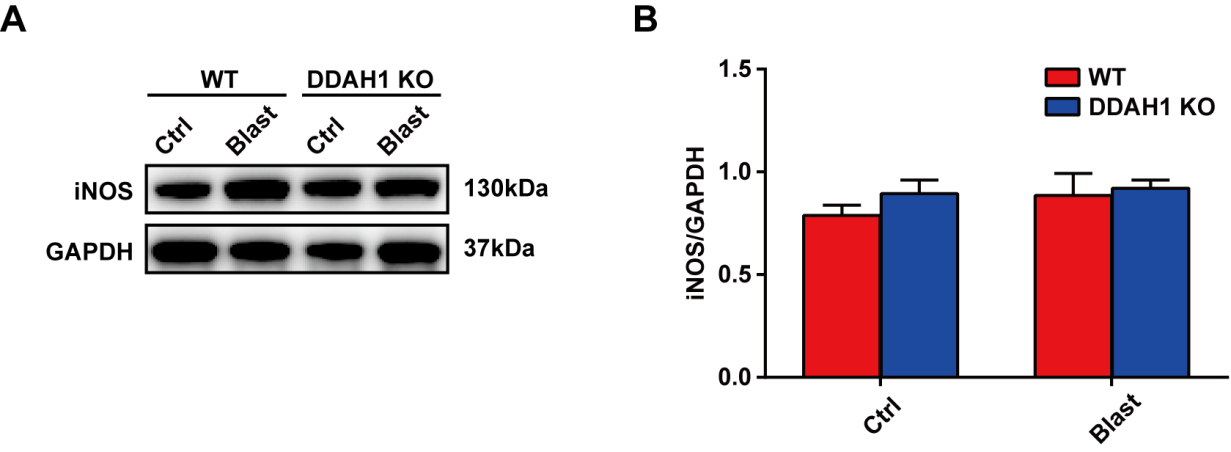


**Supplementary Figure 1. Expression of iNOS in the lung tissue after blast exposure**

**(A)** Western blot of **iNOS** in each group. **(B)** Relative density of iNOS. Data are mean ± SD.
