# Supplementary material for: DDAH1 Promotes Lung Endothelial Barrier Repair by Decreasing Leukocyte Transendothelial Migration and Oxidative Stress in Explosion-Induced Lung Injury
Source: Oxid Med Cell Longev. 2022 May 17;2022:8407635. doi: 10.1155/2022/8407635 (PMC9130000; doi:10.1155/2022/8407635)
Supplement: Supplementary Materials — Supplementary Table 1: primary antibody list. Supplementary Table 2: secondary antibody list. Supplementary Figure 1: expression of iNOS in the lung tissue after blast exposure. (a) Western blot of iNOS in each group. (b) Relative density of iNOS. Data are mean ± SD. [file 8407635.f1.zip › 8407635.f1/supplementary table.pdf]

Supplementary Table 1. Primary Antibody List

	Dilution ratio	Catalogue number	Company
IRE-1α	1:1000	#3294	cell signaling technology
MDA	1:1000	#5321	cell signaling technology
VEGF	1:1000	ab214424	Abcam
CD31	1:1000	ab222783	Abcam
MMP9	1:1000	ab38898	Abcam
Occludin	1:1000	ab216327	Abcam
Dystrophin	1:1000	ab275391	Abcam
Vimentin	1:1000	ab92547	Abcam
N-Cadherin	1:1000	#14215	cell signaling technology
ICAM1	1:1000	ab222736	Abcam
Itgal	1:1000	ab186873	Abcam
Rac2	1:1000	ab2244	Abcam
DDAH1	1:1000	ab180599	Abcam
ADMA	1:1000	#13522	cell signaling technology
eNOS	1:1000	#32027	cell signaling technology
iNOS	1:1000	#13120	cell signaling technology
GAPDH	1:5000	#2118	cell signaling technology

Supplementary Table 2. Secondary Antibody List

	Dilution ratio	Catalogue number	Company
anti-mouse secondary antibody	1:4000	#7076	cell signaling technology
anti-rabbit secondary antibody	1:4000	#7074	cell signaling technology
anti-goat secondary antibody	1:4000	ab6741	Abcam
