# Supplementary figures and images for: DDAH1 Promotes Lung Endothelial Barrier Repair by Decreasing Leukocyte Transendothelial Migration and Oxidative Stress in Explosion-Induced Lung Injury
Source: Oxid Med Cell Longev. 2022 May 17;2022:8407635. doi: 10.1155/2022/8407635 (PMC9130000; doi:10.1155/2022/8407635)

**A**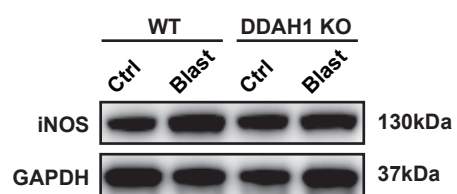**B**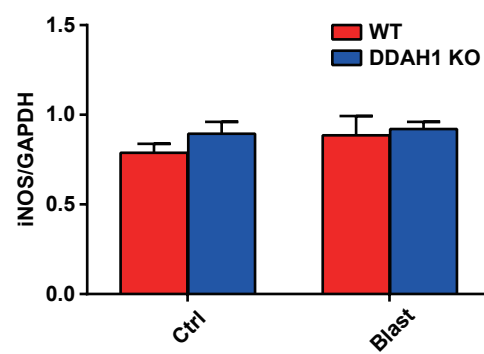

Supplement: Supplementary Materials — Supplementary Table 1: primary antibody list. Supplementary Table 2: secondary antibody list. Supplementary Figure 1: expression of iNOS in the lung tissue after blast exposure. (a) Western blot of iNOS in each group. (b) Relative density of iNOS. Data are mean ± SD. [file 8407635.f1.zip › 8407635.f1/Supplementary figure 1.pdf]
